# Practical Decision Algorithms for the Use of the Cardiovascular Polypill in Secondary Prevention in Europe

**DOI:** 10.3389/fcvm.2021.663361

**Published:** 2021-08-24

**Authors:** Lilian Grigorian-Shamagian, Klaus Edel, María Asunción Esteve-Pastor, Álvaro Aceña, Claudia Silva, Joana Delgado-Silva, Georges Ntaios, Eftychia Demerouti, Carlos Brotons

**Affiliations:** ^1^Department of Cardiology, Hospital General Universitario Gregorio Marañón, Instituto de Investigación Sanitaria Gregorio Marañón and Facultad de Medicina, Universidad Complutense de Madrid, Madrid, Spain; ^2^Centro de Investigación Biomédica en Red en Enfermedades Cardiovasculares (CIBERCV), Instituto de Salud Carlos III, Madrid, Spain; ^3^Center of Cardiovascular Diseases, Department of Cardiol. Rehabilitation, Rotenburg, Germany; ^4^Department of Cardiology, Hospital Clínico Universitario Virgen de la Arrixaca, IMIB-Arrixaca, CIBERCV, Murcia, Spain; ^5^Department of Cardiology, Hospital Universitario Fundación Jiménez Díaz, Universidad Autónoma de Madrid, Madrid, Spain; ^6^USF Cruz de Celas, Coimbra, Portugal; ^7^Department of Cardiology, Coimbra Hospital and University Centre, Coimbra, Portugal; ^8^Department of Internal Medicine, Faculty of Medicine, School of Health Sciences, University of Thessaly, Larissa, Greece; ^9^Onassis Cardiac Surgery Center, Kallithea, Greece; ^10^Sardenya Primary Health Care Center, Barcelona, Spain; ^11^Biomedical Research Institute Sant Pau, Barcelona, Spain

**Keywords:** polypill, fixed-dose combination, secondary prevention, cardiovascular disease, cerebrovascular disease

## Abstract

The main objective of cardiovascular disease (CVD) prevention is to reduce morbidity and mortality. Despite recommendations on evidence-based pharmacological treatment and lifestyle changes, the control of CV risk factors such as hypertension or dyslipidaemia is not optimal. The use of a CV polypill, including guideline-recommended drugs, as a baseline therapy, may contribute to improving risk factors control either by improving the treatment adherence or by the synergistic effect of its components. The CNIC-Polypill is the first CV polypill approved in Europe as an effective strategy for secondary prevention, which contains acetylsalicylic acid, atorvastatin (in two optional doses), and ramipril (in three optional doses) in a single pill. The present practical clinical document aims to provide a guide for patient management after an acute coronary syndrome (ACS) or with chronic CVD (CCVD) with a strategy based on the CNIC-Polypill, also considering the need to add other therapies for a personalized treatment. The most suitable clinical scenarios for the CNIC-Polypill use are discussed: (a) in patients after an ACS at discharge, (b) in patients with CCVD (chronic coronary syndrome, stroke, or peripheral artery disease) with uncontrolled low-density lipoprotein cholesterol (LDL-c) and/or blood pressure levels and (c) in patients with CCVD with well-controlled risk factors to simplify treatment and reduce polypharmacy in the context of CCVD prevention.

## Introduction

Cardiovascular disease (CVD) is a global condition that represents the main cause or mortality and morbidity worldwide (1). In Europe, CVD caused 4.1 million deaths in 2017, which represents 47% of all deceases, and it is also the principal cause of mortality in subjects <70 years old (2).

The main objective of CVD prevention is to reduce morbidity and mortality (3). Lifestyle modifications and pharmacological treatment are recommended by clinical guidelines to achieve this goal (3). In the particular case of secondary prevention, current guidelines recommend at least the use of the following drug classes (3–7): low-dose acetylsalicylic acid (ASA; 75–100 mg), statins and angiotensin-converting enzyme inhibitors (ACEIs) or angiotensin II receptor blockers (ARBs). Additional drugs might be added for a better risk factor control such as ezetimibe and/or proprotein convertase subtilisin/kexin type 9 inhibitors (PCSK9i), diuretics, beta blockers (BB) or calcium channel blockers (CCBs), and a P2Y12 inhibitor.

Despite recommendations, the EUROASPIRE survey showed that most coronary patients failed to meet the risk factor targets (8). In this context, the polypill concept emerged almost two decades ago, aiming to minimize the problems associated with polypharmacy and low adherence in CV patients (9). However, nowadays, the role of a polypill in CV prevention has evolved, and it might be considered as a baseline treatment in association with other drugs to achieve risk factor control and therefore reduce CV risk according to the recent European Society of Cardiology (ESC) guidelines (5). There has been accumulated evidence in the last years reinforcing this concept. Both randomized clinical trials (RCTs) and real-world studies have shown that the improvement in the control of CV risk factors (e.g., systolic blood pressure [BP] and low-density lipoprotein [LDL] cholesterol) is greater among subjects using the CV polypill than those in usual care or with medications administered separately (10–16). Additionally, one large-scale study demonstrated a decrease in CV events (i.e., hospitalization for acute coronary syndrome (ACS), fatal myocardial infarction, sudden death, heart failure (HF), coronary artery revascularization procedures, and non-fatal and fatal stroke) in the polypill group compared with the group of patients on usual care (17). These outcomes are currently being assessed in elderly patients with a recent myocardial infarction with the CNIC-Polypill formulation in the SECURE trial (https://clinicaltrials.gov/ct2/show/NCT02596126).

The greater efficacy and effectiveness of the CV polypill vs. usual care have been largely attributed to enhanced adherence (18–20). Indeed, poor adherence to prescribed medications is associated with higher risk of CV events, increased mortality and risk of hospitalization; and it is a particular challenge in the elderly population (21–24). However, it has been recently shown that, besides adherence, the improvement in the lipid profile with the CV polypill might also be due to a synergistic effect within the components (statin and ACEI) leading to a further reduction in terms of LDL-c equivalent to doubling the dose of the statin in monotherapy (25). Finally, although it has been suggested that CV polypills could potentially help to overcome therapeutic inertia (26, 27), full implementation in real-world clinical practice has been shown to be inhibited by prescription inertia (9). A recent study even showed that the rates of therapeutic inertia were higher in patients prescribed triple fixed-dose combinations (FDC) of antihypertensives than in usual care (28). Despite this, the BP control rates at the end of the study were higher among those in the FDC arm, showing that, even when therapeutic inertia is present, the polypill strategy confers an advantage regarding CV risk factors control over the traditional multipill approach.

Different formulations of CV polypills are currently available, all of them including a statin, one or more antihypertensive medications (ACEI/ARB/thiazide/BB/CCB) and sometimes an antiplatelet agent (usually ASA) (9). The first CV polypill approved and marketed in Europe for the secondary prevention of CVD was the CNIC-Polypill, which contains ASA 100 mg, atorvastatin (20 or 40 mg) and ramipril (2.5, 5, or 10 mg) (9, 29).

Recently, an article from Coca et al. proposed algorithms to guide doctors to start or switch to the most appropriate CNIC-Polypill dosage based on the baseline hypertension grade in patients at high risk or with established CVD (26). In line with this, here, we provide practical decision algorithms to facilitate the use of the CNIC-Polypill strategy in patients with ACS and patients with an established CVD, including chronic coronary syndrome (CCS), stroke or peripheral artery disease (PAD). Finally, we propose the necessary steps to reduce polypharmacy in patients with established CVD through the use of the CNIC-Polypill.

## Development of the Algorithms

An academic expert panel of nine members was selected from different countries, namely, Spain, Germany, Portugal, and Greece. The experts were chosen based on their expertise in cardiology, internal medicine, or family medicine. A coordinating committee of two experts was formed, one in charge of the ACS algorithm and the other the CCS algorithm. The remaining experts were organized in balanced working groups where each participant was involved in a single algorithm.

As a first step, each coordinator developed a draft of the corresponding algorithm that was further sent to the related working group together with a written questionnaire with clinically relevant questions regarding current pharmacological treatments and country-specific guidelines or recommendations. Each coordinator gathered the information and interviewed each participant to prepare a document with clinical questions that included potential statements and conclusions. In a second online meeting, each working group debated the algorithm based on the contributions and amendments made. All the agreed recommendations and conclusions were included in a new version of the algorithms. A subsequent validation round was performed where each algorithm was approved as the final version.

## What is the Patient Profile Suitable for the CNIC-Polypill?

Overall, we defined as suitable for secondary prevention those patients with an established atherosclerotic CVD, namely, a coronary artery disease (i.e., acute myocardial infarction [AMI], unstable angina and CCS), a cerebrovascular disease (i.e., atherothrombotic stroke, transient ischemic attack [TIA] and lacunar infarction, excluding cardioembolic or haemorrhagic stroke) or PAD.

The therapeutic goals and the corresponding armamentarium for secondary CVD prevention are described in detail in the different ESC clinical practice guidelines, consensus, and position papers (available online at https://www.escardio.org/Guidelines). Moreover, national clinical guidelines or formal consensus documents from a particular EU country may be used (searchable online through https://www.escardio.org/The-ESC/Member-National-Cardiac-Societies), as they may have adapted the content and recommendations for the local setting, such as the substitution of a drug that is unavailable locally, or different rehabilitation and prevention interventions depending on the healthcare infrastructure or insurance plan type.

First and foremost, it is essential to identify those patients who would benefit from the use of the CNIC-Polypill as a baseline pharmacological treatment to control CV risk factors (CVRFs). This requires a personalized approach; and the choice must be therefore based on the particular patient's profile, understood as the combination of the clinical history and drug tolerability. On this basis, the proposed pharmacological intervention of the present guidance document has been divided into (a) the control of CVRF after an acute event (i.e., ACS) and (b) the maintenance or continuation of CVRF management for chronic conditions (defined as CCS, stroke or PAD) in outpatient clinics, rehabilitation institutions or primary care. In all cases, the purpose is to attain and maintain the recommended levels of LDL-c and BP with the CNIC-Polypill through the choice of the best formulation (different doses of atorvastatin and ramipril) and to help in adding concomitant medications if required.

## Secondary Prevention After an Acute Coronary Syndrome

We include here ACS manifesting as MI or unstable angina. Management typically takes place in the inpatient setting, and the therapeutic approach has been divided into early intervention (during in-hospital stay) and that at the moment of hospital discharge or referral for hospital-based or outpatient cardiac rehabilitation.

On admission to the hospital, and immediately after the index episode, the first aim is the patient's clinical stabilization and the prevention of complications. During this hospital stay and early-phase treatment, the CNIC-Polypill is not considered appropriate because of the potential existence of haemodynamic instability and thus the requirement of complex additional/concomitant treatments or invasive procedures in high-risk patients. Thus, the CNIC-Polypill is preferentially indicated at the moment of discharge or referral for hospital-based or outpatient cardiac rehabilitation.

According to the ESC guideline (5, 30), and besides coronary revascularization when indicated, routine pharmacological therapies comprise dual antiplatelet therapy (DAPT; a combination of aspirin and a P2Y_12_ inhibitor), ACEI/ARB, BB and a mineralocorticoid receptor antagonist (MRA) in patients with HF or left ventricle (LV) dysfunction, oral anticoagulant in some cases and starting (or adjusting) high-intensity statin therapy (5). Lipid-lowering therapy must be initiated as soon as possible after admission, and the recommended lipid targets (both achievement and maintenance) are set to LDL-c <55 mg/dl in the latest 2019 ESC guidelines (31). Moreover, a first control of LDL-c values should be carried out after 4–6 weeks from discharge to adapt the therapeutic regimen accordingly (31).

**Figure 1:**
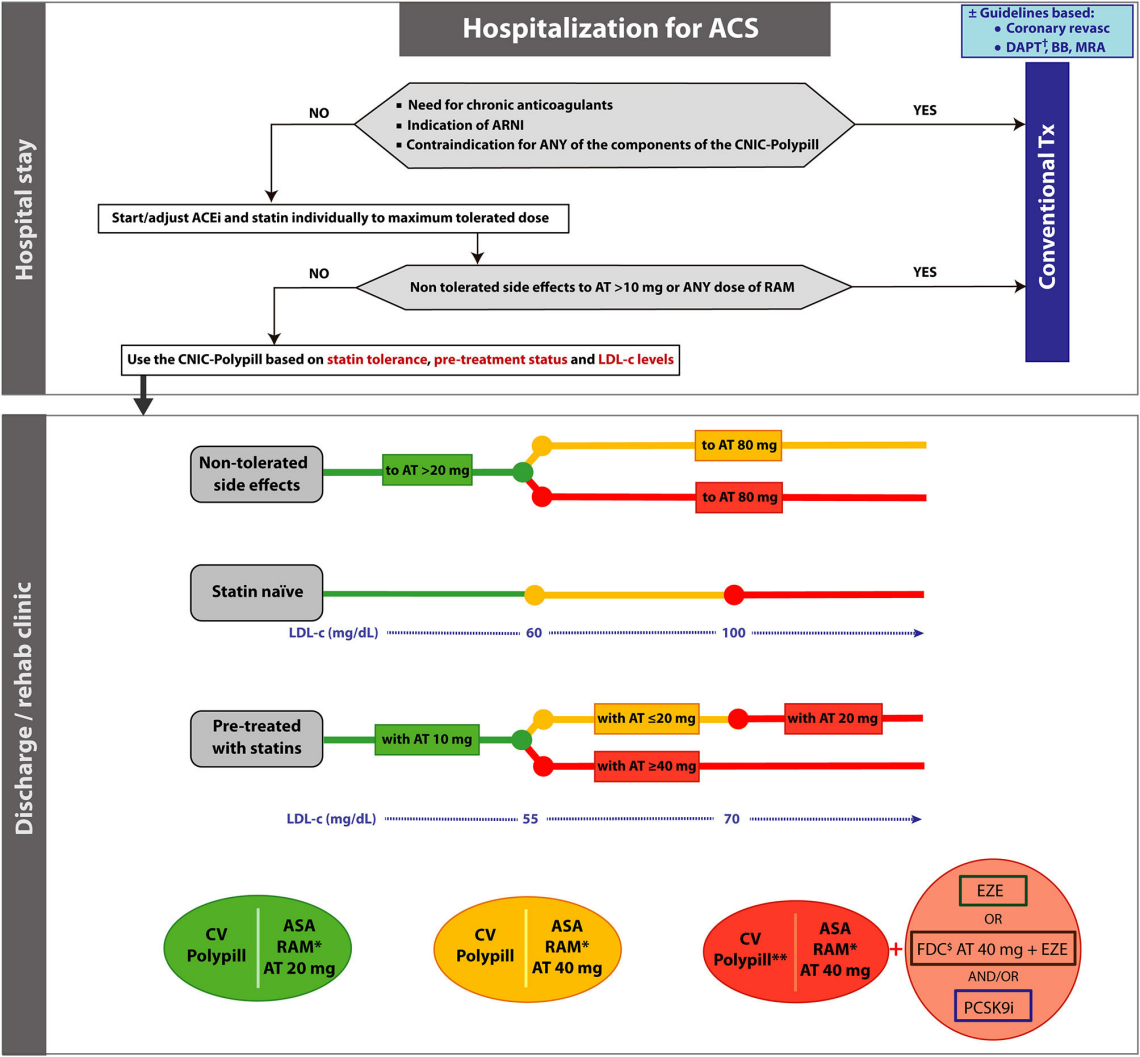
Algorithm showing the steps and options to switch patients hospitalized for an acute coronary syndrome to the CNIC-Polypill strategy. Note: The colored balls represent the appropriate formulation of the CNIC-Polypill according to the colored lines of the algorithm. ^†^Select P2Y_12_ inhibitor in addition to the CNIC-Polypill. ^*^Dose adjusted to BP levels, previous ACEI/ARB dose and renal function. ^**^Reassess in 3–4 weeks after discharge and readjust the dosage, consider adding A40 or ezetimibe and/or a PCSK9i. ^$^Use only if the patient does not develop side effects to atorvastatin 80 mg (or equivalent doses of another statin). ACEI, angiotensin-converting enzyme inhibitor; ACS, acute coronary syndrome; ARB, angiotensin II receptor blocker; ARNI, angiotensin receptor neprilysin inhibitor; ASA, acetylsalicylic acid; AT, atorvastatin; BB, beta blocker; BP, blood pressure; DAPT, dual platelet therapy; EZE, ezetimibe; FDC, fixed-dose combination; LDL-c, low-density lipoprotein cholesterol; PCSK9i, proprotein convertase subtilisin/kexin type 9 inhibitor; RAM, ramipril; Tx, treatment.

The suitability of the CNIC-Polypill at discharge/rehabilitation depends on the patient's profile (Figure 1). Firstly, conventional treatment, as recommended in current clinical guidelines, is the preferred option vs. the CNIC-Polypill in the case of any of the following instances:

1) Need for long-term chronic oral anticoagulation in patients with previous treatment, with atrial fibrillation (AF), mechanical valve, pulmonary, and/or venous thromboembolism.
2) Indication for an angiotensin receptor neprilysin inhibitor (ARNI) in patients with chronic symptomatic HF with reduced ejection fraction (HFrEF).
3) Contraindication of any of the individual components of the CNIC-Polypill, i.e., either statins, ACEI, or ASA.

When all the above-mentioned conditions have been ruled out, the initiation of the treatment with the CNIC-Polypill may be considered and adjusted based on the following general rules (Figure 1): (1) for atorvastatin, depending on statin tolerance, prior pharmacological prescriptions and LDL-c levels; (2) for ramipril, based on BP levels, previous ACEI/ARB dose and kidney function (32); and (3) although the low-dose ASA (100 mg) does not require further dose adjustment, a P2Y_12_ inhibitor must be added to the CNIC-Polypill in all cases to comply with DAPT.

Since the selection of atorvastatin dose may be the cornerstone of the CNIC-Polypill prescription in this setting, below, we present a simple decision-making algorithm based on several parameters presented separately for ease of understanding.

### According to the Statin Tolerance Levels

Muscle toxicity (including myopathy and rhabdomyolysis) and liver enzyme elevations are the most well-recognized effects of statins, with 10–15% of patients reporting statin-associated muscle symptoms, and alanine aminotransferase (ALT) elevation may be observed in 0.5–2% of the subjects (31).

The risk of statin-induced muscle toxicity is more likely at high doses, and it has been estimated that about 60% of statin-related rhabdomyolysis cases are related to drug interactions (33). While atorvastatin 10–20 mg is considered a moderate-intensity statin (LDL-c reduction 30–49%), doses of 40 to 80 mg may be considered high-intensity statins (LDL-c reduction ≥50%) (34).

In the proposed algorithm (Figure 1), the options in patients according to the tolerance to statins are as follows:

1. If the patient develops side effects to doses of atorvastatin higher than 20 mg or equivalent dose of another statin, choose the CNIC-Polypill containing atorvastatin 20 mg (green option in Figure 1). Consider adding ezetimibe and/or PSCK9i to reach the goals.
2. If the patient develops side effects to doses of atorvastatin 80 mg (or equivalent dose of another statin) but tolerates lower doses, there are two options in order to achieve the target level of LDL-c:
  a. Use the CNIC-Polypill with 40 mg of atorvastatin alone (orange option in Figure 1) when this is sufficient to achieve the target.
  b. Use the CNIC-Polypill with 40 mg of atorvastatin and consider adding ezetimibe and/or a PSCK9i (red option in Figure 1) to reach the LDL-c target level (i.e., <55 mg/dl) (31).

The key concept here is that the primary goal is to reduce the relative CV risk through the reduction of the baseline LDL-c regardless of the therapeutic intervention used, namely, a high-intensity strategy, involving statins and other blood lipid-lowering drugs when needed (35).

### Statin Naïve Patients

In patients not previously on statin therapy before hospitalization, the CNIC-Polypill formulation must be chosen based on the LDL-c levels at discharge. Based on the expected LDL-c reduction with a specific dose of atorvastatin, the options are as follows:

1. In cases where levels are below 60 mg/dl, use the atorvastatin 20 mg CNIC-Polypill formulation (green option in Figure 1), as this dose will allow reaching the target of <55 mg/dl as recommended in the ESC clinical guidelines (31).
2. If the LDL-c level is between 60 and 100 mg/dl, use the atorvastatin 40 mg CNIC-Polypill formulation (orange option in Figure 1).
3. If the LDL-c level is over 100 mg/dl, go directly to atorvastatin 40 mg CNIC-Polypill option and add ezetimibe (or use an FDC of atorvastatin 40 mg plus ezetimibe) and/or a PSCK9i (red option in Figure 1). Although it is also an option to use atorvastatin 80 mg as the first choice, the rationale behind the use of CNIC-Polypill atorvastatin 40 mg instead is the synergistic effect between atorvastatin and ramipril observed with the use of the CNIC-Polypill, which results in 7% additional LDL-c reduction compared with statin in monotherapy, which is equivalent to doubling the dose of the statin (estimated to correspond to a 6–8% reduction) (25). With this alternative CNIC-Polypill, there is an added value when it comes to a simplified drug regimen that can increase adherence in patients starting long-term treatment and, ultimately, improve risk factor control.

### Patients Previously Treated With Statins

In patients already on statin therapy prior to hospitalization, the CNIC-Polypill formulation must be chosen based on the LDL-c levels and the previous dose of statin.

1. In patients on a previous low dose of atorvastatin or equivalent (i.e., 10 mg) and LDL-c levels up to 55 mg/dl, it is reasonable to switch to a double dose of atorvastatin, that is, 20 mg (green option in Figure 1).
2. If the patient was treated with atorvastatin or equivalent at doses lower or equal to 20 mg and has LDL-c levels between 55 and 70 mg/dl, switching to the CNIC-Polypill formulation with 40 mg of atorvastatin should be considered (orange option in Figure 1).
3. For those treated with atorvastatin or equivalent at doses lower or equal to 20 mg and LDL-c levels above 70 mg/dl, switch to the option of the CNIC-Polypill with 40 mg of atorvastatin and consider adding ezetimibe (or use an FDC of atorvastatin 40 mg plus ezetimibe) and/or a PSCK9i (red option in Figure 1).
4. In all patients on atorvastatin or equivalent at doses higher or equal to 40 mg and out of LDL-c target (i.e., >55 mg/dl), directly use the CNIC-Polypill with atorvastatin 40 mg and add ezetimibe (or use an FDC of atorvastatin 40 mg plus ezetimibe) and/or a PSCK9i (red option in Figure 1).

## Secondary Prevention in Chronic Cardiovascular Disease

**Figure 2:**
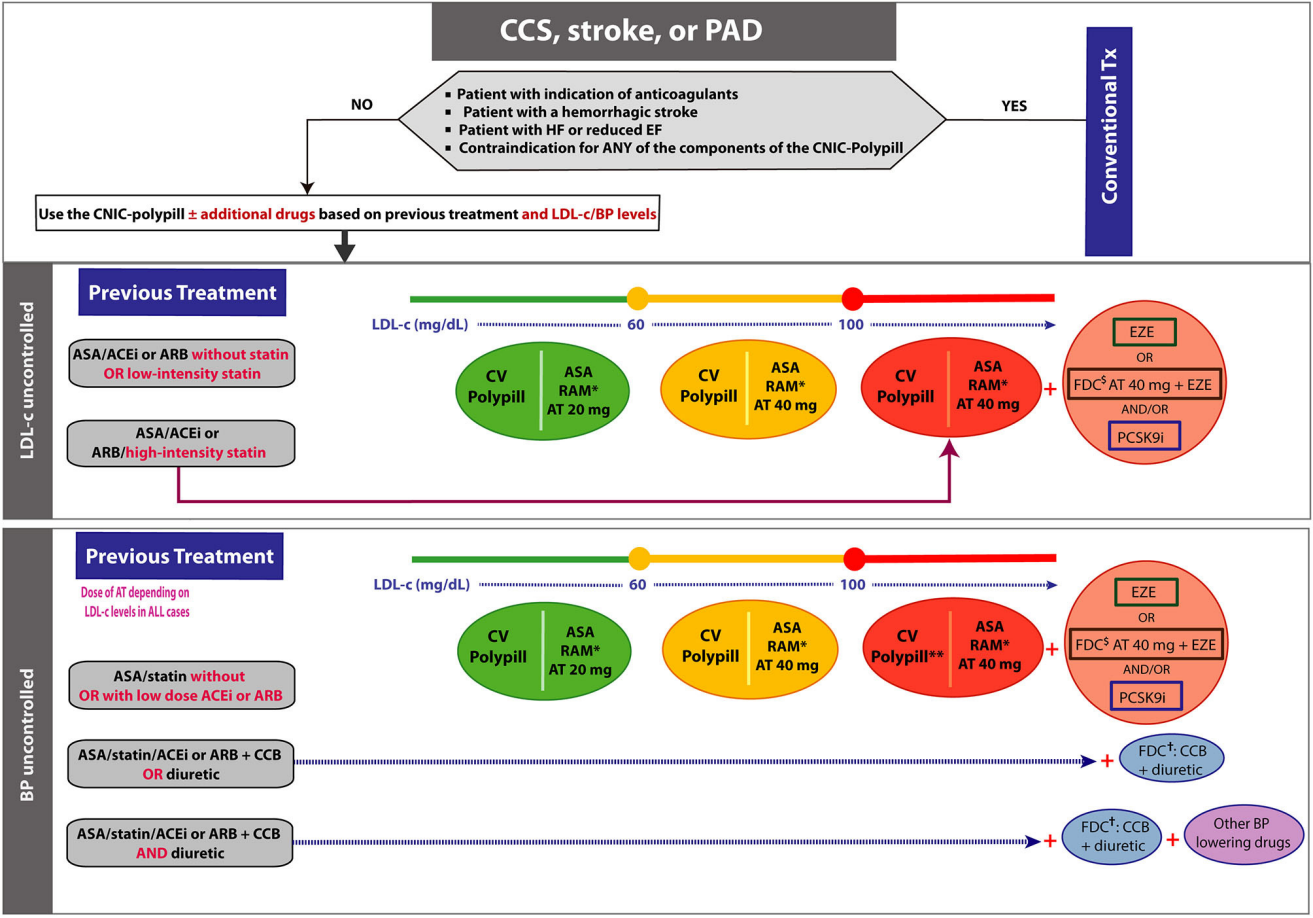
Algorithm showing the steps and options to switch patients treated for chronic coronary syndromes to the CNIC-Polypill strategy. Note: The colored balls represent the appropriate formulation of the CNIC-Polypill according to the colored lines of the algorithm. ^*^Dose adjusted to BP levels, previous ACEI/ARB dose and renal function. ^**^See above for additional treatment of high LDL-c levels. ^$^Use only if the patient does not develop side effects to atorvastatin 80 mg (or equivalent doses of another statin). ^†^If the combination is available. ACEI, angiotensin-converting enzyme inhibitor; ARB, angiotensin II receptor blocker; ASA, acetylsalicylic acid; AT, atorvastatin; BP, blood pressure; CCB, calcium channel blocker; CCS, chronic coronary syndrome; EZE, ezetimibe; FDC, fixed-dose combination; LDL-c, low-density lipoprotein cholesterol; PAD, peripheral artery disease; PCSK9i, proprotein convertase subtilisin/kexin type 9 inhibitor; RAM, ramipril; Tx, treatment.

**Figure 3:**
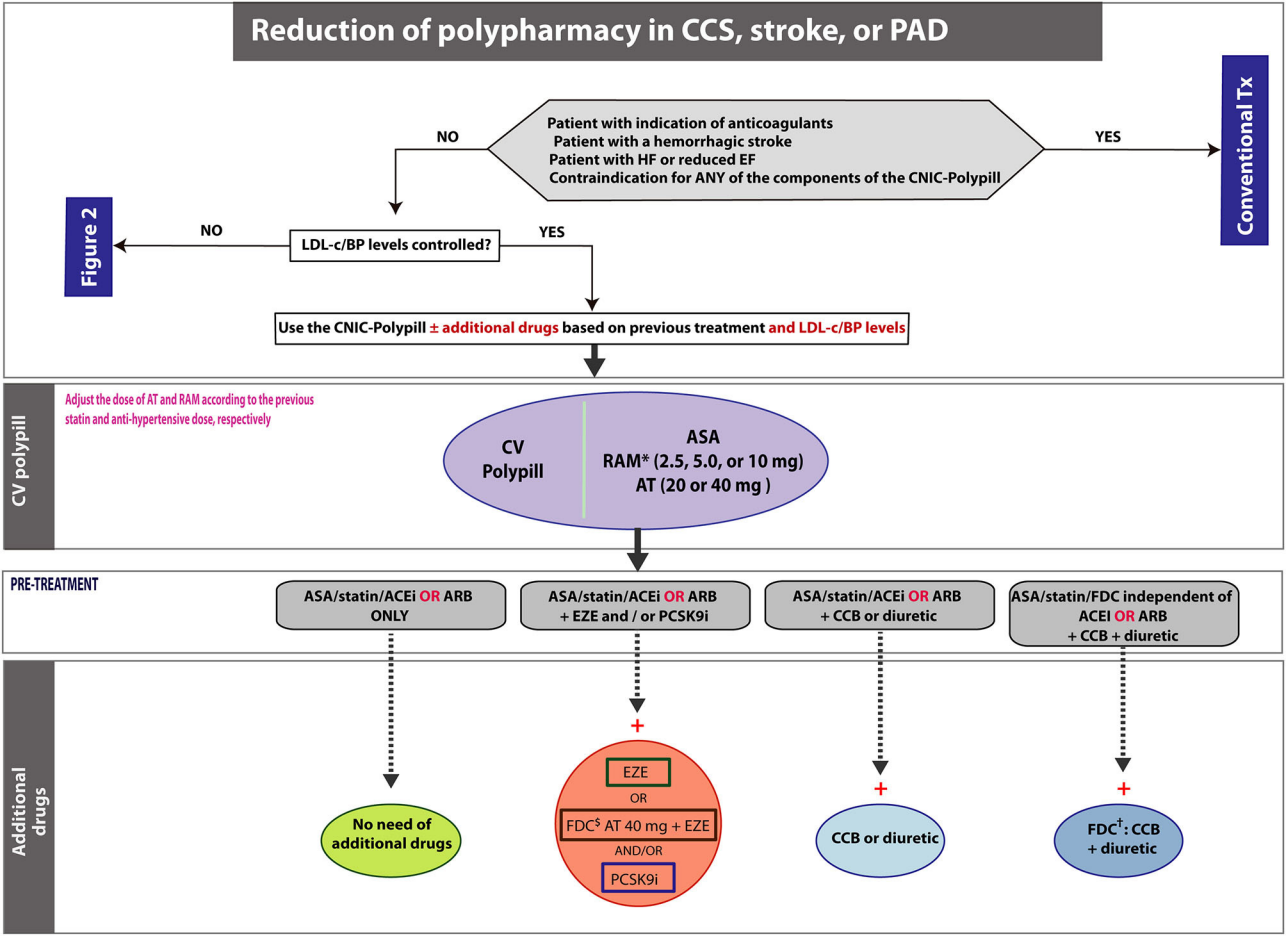
Algorithm showing the steps and scenarios to reduce the pill burden with the CNIC-Polypill in patients treated for chronic CVD. Note: ^*^Dose adjusted to previous ACEI/ARB dose and renal function. ^$^Use only if the patient does not develop side effects to atorvastatin 80 mg (or equivalent doses of another statin). ^†^If the combination is available. ACEI, angiotensin-converting enzyme inhibitor; ARB, angiotensin II receptor blocker; ASA, acetylsalicylic acid; AT, atorvastatin; BP, blood pressure; CCB, calcium channel blocker; CCS, chronic coronary syndrome; EZE, ezetimibe; FDC, fixed-dose combination; LDL-c, low-density lipoprotein cholesterol; PAD, peripheral artery disease; PCSK9i, proprotein convertase subtilisin/kexin type 9 inhibitor; RAM, ramipril; Tx, treatment.

This section proposes decision algorithms to be used by clinicians who want to use the CNIC-Polypill in patients with CCS, established atherothrombotic stroke or PAD (Figure 2). Moreover, we provide a decision algorithm to switch to the polypill strategy in well-controlled patients with polypharmacy to reduce the pill burden (Figure 3). In all cases, and in the first instance, the patient must be a candidate to switch to the CNIC-Polypill strategy only if the following conditions are fulfilled (see above).

Patients treated for established CVD have been carefully assessed and monitored during the time that they have been on treatment so that alleged side effects or intolerances are known. For instance, in the event of a high risk of gastrointestinal bleeding in patients on ASA (either as monotherapy or DAPT), it is recommended to add a proton pump inhibitor (PPI) (36). Moreover, all patients should already be on statins, thus requiring the evaluation of the liver function and muscular affection if the prescribed dose was high; and since many patients have conditions requiring an ACEI (e.g., HF, hypertension or diabetes), the monitoring of the kidney function is mandatory.

Once the patient is considered as a candidate to switch to the CNIC-Polypill, the initial recommendations will depend on the previous pharmacological treatment and whether the patient's BP and LDL-c are well-controlled, thus requiring in certain circumstances the addition of concomitant drugs (Figure 2). Indeed, suboptimal CV risk factor control is common in secondary CV prevention, as reported by the EUROASPIRE (European Action on Secondary and Primary Prevention through Intervention to Reduce Events) surveys (8). In this international study conducted in patients with coronary artery events or interventions, 95% of subjects were prescribed BP-lowering drugs, but only 54% were at or below the recommended targets 6 months after the event (8). Likewise, among the 84% that were on lipid-lowering drugs, up to 68% of them were not at target LDL-c <70 mg/dl (8). Another European cross-sectional study conducted in 973 patients with established CVD (coronary heart disease (CHD) and stroke) being attended in primary care showed that LDL-c target value below 70 mg/dl was achieved in about 23% of patients, and about 75% of patients reached the <140/90 mmHg target for BP (37).

### Patients With Suboptimal Low-Density Lipoprotein Cholesterol Levels

We define here as optimal LDL-c levels those recommended by the latest 2019 ESC guidelines for the management of dyslipidaemias and of CCS, that is, <55 mg/dl (<1.4 mmol/L) (31, 36).

Despite guidelines recommending high-intensity statins, the EUROASPIRE study reported that, in Europe, about 10% of patients with established CHD did not have a statin prescription at discharge, about 62.4% started on low- or medium-intensity statins, and this proportion only increased to 67.3% after 1 year (38, 39). This suboptimal prescription is of concern if we take into account that the risk of recurrent CV events, CV mortality and also all-cause mortality decrease in parallel with the potency of the statin used (39, 40).

The introduction of the CNIC-Polypill in patients with uncontrolled LDL-c levels will depend on both the previous therapeutic regimen and the baseline LDL-c values, namely, whether they are close or below the target (i.e., 60 mg/dl) or above, in turn divided into those with levels between 60 and 100 mg/dl and those with high levels >100 mg/dl (Figure 2). The key message is that all patients not treated with statins or with non-high intensity statins must be up-titrated to a higher potency statin. The following clinical scenarios are proposed:

#### Patients on Previous Treatment With ASA/ACEI or ARB Without Statin or With Low-Intensity Statins (Atorvastatin <20 mg or Equipotent Statins)

a. In statin-naïve patients with LDL-c levels very close to the goal (<60 mg/dl), the CNIC-Polypill with 20 mg of atorvastatin is a good option to start with (green option in Figure 2) in order to reach the target. An additional advantage of starting with the 20-mg atorvastatin CNIC-Polypill is that patients who are actually non-adherent to the previously prescribed low-dose statin will have the opportunity to optimize their LDL-c levels before increasing the dose to 40 mg. Moreover, in particular populations of patients such as elderly subjects, with previous side effects to high-intensity statins or bed-bound, starting with the CNIC-Polypill atorvastatin 20 mg is a good option.
b. If the baseline LDL-c level is between 60 and 100 mg/dl, then the CNIC-Polypill with 40 mg of atorvastatin would be more appropriate to reach the target (orange option in Figure 2).
c. Finally, for those patients with high baseline LDL-c levels above 100 mg/dl, the best option would be to use the CNIC-Polypill with 40 mg of atorvastatin and add ezetimibe (or use an FDC of atorvastatin 40 mg plus ezetimibe) and/or a PSCK9i (red option in Figure 2).

#### Patients on Previous Treatment With ASA/ACEI or ARB With a High-Intensity Statin (or Equipotent to 80 mg of Atorvastatin)

In this case, the option would be to directly use the CNIC-Polypill 40 mg plus ezetimibe (or use an FDC of atorvastatin 40 mg plus ezetimibe) and/or a PSCK9i (red option in Figure 2). As stated in the ACS algorithm, and beyond the fact that atorvastatin 80 mg is not always well-tolerated, the reported synergistic effect of atorvastatin 40 mg and ramipril would be equivalent to doubling the dose of the statin (therefore 80 mg) (25).

### Patients With Suboptimal Blood Pressure Levels

In cases where BP is poorly controlled (i.e., >130/80 mmHg), the introduction of the CNIC-Polypill will depend on both the previous antihypertensive treatment and also the LDL-c levels (Figure 2). For patients on ACEIs or ARBs, the switch to the CNIC-Polypill will require an adjustment of the ramipril dose (i.e., 2.5, 5.0, or 10 mg) depending on the hypertension grade and based on the approximate corresponding equivalent effective daily doses. A guideline on how to switch polypill for CVD prevention in patients with hypertension has been previously published and can be used for additional details (26). Moreover, the atorvastatin dose must be adjusted to the baseline LDL-c levels and follow the recommendations of the previous section.

The three following scenarios can be found in clinical practice:

#### Patients on ASA/Statin but Not on BP-Lowering Treatment or Low Doses of ACEI or ARB

The switch to the CNIC-Polypill is an option that will only require to use of the formulation with the adequate dose of ramipril and atorvastatin (or additional lipid-lowering drugs) based on the baseline LDL-c levels (i.e., close to target or <60 mg/dl, between 60 and 100 or >100 mg/dl).

#### Patients on ASA/Statin/ACEI or ARB Plus a CCB or a Diuretic

In this case, and besides the dose adjustments mentioned earlier for ramipril and atorvastatin, the patient will need to add an FDC of CCB and a diuretic to the chosen CNIC-Polypill formulation.

#### Patients on ASA/Statin/ACEI or ARB Plus a CCB and a Diuretic

For these subjects, the CNIC-Polypill, chosen based on the required ramipril and atorvastatin doses, must be combined with an FDC of CCB plus a diuretic, and the clinician needs to consider intensifying treatment with an additional BP-lowering drug excluding another renin–angiotensin–aldosterone system (RAAS) blocker as it is contraindicated in the summary of product characteristics (SmPC) of ramipril.

## How to Reduce Polypharmacy in Patients With Well-Controlled Blood Pressure and Low-Density Lipoprotein Cholesterol

In this section, we describe how to implement the CNIC-Polypill in the context of patients whose BP and LDL-c levels are well-controlled but are treated with five or more drugs (Figure 3). Notably, polypharmacy lead not only to low adherence to preventive CV treatment but also to suboptimal prescribing and increased costs (41). The most common type of adverse drug events (ADEs) among the elderly are unintentional overdoses, which in more than half of cases involve five or more concomitant medications and in 65.7% of patients require hospitalization (42).

**Figure 4:**
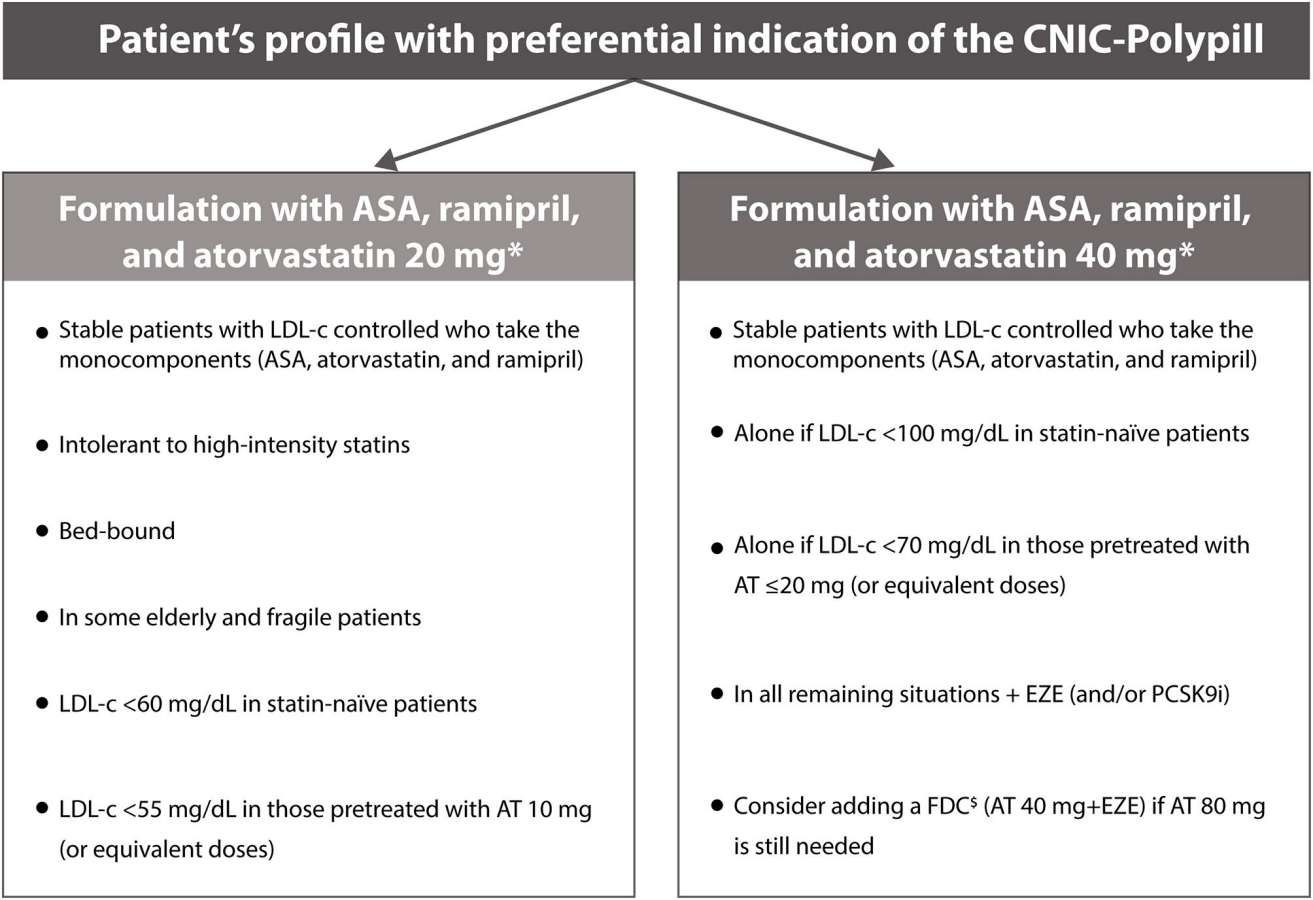
Summary of the adequate profile of patients who are considered candidates to switch to the CNIC-Polypill. Note: ^*^Or equivalent doses of the monocomponents. ^$^Use only if the patient does not develop side effects to atorvastatin 80 mg (or equivalent doses of another statin). ACS, acute coronary syndrome; AT, atorvastatin; CV, cardiovascular; EZE, ezetimibe; FDC, fixed-dose combination; LDL-c, low-density lipoprotein cholesterol; PCSK9i, proprotein convertase subtilisin/kexin type 9 inhibitor.

In view of the complexity of patients requiring CVD secondary prevention, the polypill strategy may be particularly advantageous in patients on polypharmacy. When switching to the CNIC-Polypill, the most appropriate dose of atorvastatin (20 or 40 mg) and ramipril (2.5, 5.0, or 10.0 mg) must be chosen according to the previous doses that allowed optimal control of LDL-c levels and BP, respectively. Additional guidance on the patient's profile for each dose is summarized in Figure 4.

The following clinical scenarios are proposed (Figure 3):

### Patients Treated With ASA/Statin/ACEI or ARB Only (From 3 to 1 Pill)

In patients receiving three independent drug classes, the CNIC-Polypill can be used as a single treatment and will result in the reduction to only one pill.

### Patients Treated With ASA/Statin/ACEI or ARB Plus Additional Lipid-Lowering Drugs (From 4/5 to 2/3 Pills)

In patients receiving three independent drug classes and also other lipid-lowering agents (i.e., ezetimibe and/or PCSK9i), the adequate CNIC-Polypill can be used combined with ezetimibe (which will result in the reduction to two pills) and/or a PCSK9i (which will result in the reduction to two or three pills). An additional option would be to associate an FDC of atorvastatin 40 mg plus ezetimibe with the CNIC-Polypill, which would also lead to the reduction from four to two pills.

### Patients Treated With ASA/Statin/ACEI or ARB Plus Additional BP-Lowering Drugs (From 3/4/5 to 2 Pills)

In patients receiving three independent drug classes and a CCB or a diuretic to further control BP, the CNIC-Polypill can be used, combining it with a CCB or a diuretic, thus reducing from four to two pills. In the event that the patient was treated with one therapeutic class FDC of ACEI/ARB plus a CCB or a diuretic, the reduction would still be from three to two tablets.

If the patient was on three independent drug classes and both a CCB and a diuretic, the CNIC-Polypill can be combined with an FDC of a CCB plus a diuretic, thus reducing from five to two pills.

In patients receiving ASA, a statin and an FDC of three antihypertensive drugs (i.e., composed of an ACEI or an ARB, a CCB and also a diuretic) to further control BP, the CNIC-Polypill can be used in combination with an FDC of a CCB plus a diuretic, thus reducing from three to two pills. Please note that the use of an FDC with three antihypertensive components may not be common in some European countries, meaning that the benefit of the CNIC-Polypill will lower the pill burden from five (if multiple independent pills) or four (if using a two-drug FDC of ACEI/ARB plus a CCB or a diuretic) to two pills.

## Limitations

Several limitations should be borne in mind when planning to or implementing the algorithms. Firstly, they are focused on the CNIC-Polypill and, thus, not applicable to other CV polypills with a different number of components and/or doses. Secondly, the maximum dose of atorvastatin currently available in the CV polypill is 40 mg. As such, for patients already on that (or equivalent) dose who are not at LDL-c target, clinicians may choose to intensify the treatment with an additional 20- or 40-mg atorvastatin tablet. However, they must consider that the combination of atorvastatin and ramipril has a synergistic effect equivalent to doubling the statin dose to, therefore, 80 mg (25). Thirdly, although the CV polypill strategy reduces the number of drugs and improves adherence compared with the multipill therapy (20), non-adherence would entail the therapeutic failure of three different drugs. Finally, the possible requirement of additional drug classes to achieve therapeutic targets may represent an additional barrier if non-adherence is present. Therefore, it is crucial that the physician establishes effective communication with the patients so that they perceive the CNIC-Polypill as a base treatment essential to control different risk factors simultaneously.

## Conclusions

This document describes how and why the CNIC-Polypill is a valid strategy that can be implemented in the following different secondary CV prevention scenarios: (a) at discharge after hospitalization because of an ACS; (b) in patients with a CCS, stroke or PAD with uncontrolled LDL-c and/or BP levels; and (c) to simplify treatment and reduce the pill burden when polypharmacy is an issue in the context of chronic CVD. For each clinical situation, the participating panel of three different specialties is key in managing these patients—namely, cardiologists, internal medicine specialists and general practitioners—identifying the most suitable patients and offering the guidelines to manage them efficiently with the CNIC-Polypill strategy.

## Data Availability Statement

The original contributions presented in the study are included in the article/supplementary material, further inquiries can be directed to the corresponding author/s.

## Author Contributions

LG-S and CB drafted the initial version of the manuscript and were the scientific coordinators of the study. All authors have contributed to develop the treatment algorithms and made critical revisions of the manuscript.

## Conflict of Interest

LG-S and CB received a grant from Ferrer Internacional, S.A. for coordination. The remaining authors declare that the research was conducted in the absence of any commercial or financial relationships that could be construed as a potential conflict of interest.

## Publisher's Note

All claims expressed in this article are solely those of the authors and do not necessarily represent those of their affiliated organizations, or those of the publisher, the editors and the reviewers. Any product that may be evaluated in this article, or claim that may be made by its manufacturer, is not guaranteed or endorsed by the publisher.
